# Effect of a multifactorial interdisciplinary intervention on mobility-related disability in frail older people: randomised controlled trial

**DOI:** 10.1186/1741-7015-10-120

**Published:** 2012-10-15

**Authors:** Nicola Fairhall, Catherine Sherrington, Susan E Kurrle, Stephen R Lord, Keri Lockwood, Ian D Cameron

**Affiliations:** 1 Rehabilitation Studies Unit, Sydney Medical School, The University of Sydney, Ryde, 2112, Australia; 2 The George Institute for Global Health, The University of Sydney, Sydney, 2000, Australia; 3 Curran Ageing Research Unit, Division of Rehabilitation and Aged Care, Hornsby Ku-ring-gai Health Service, Hornsby, NSW, Australia; 4 Falls and Balance Research Group, Neuroscience Research Australia, University of New South Wales, Sydney, 2031, Australia

**Keywords:** exercise, frail elderly, International Classification of Functioning, Disability and Health, RCT

## Abstract

**Background:**

Interventions that enhance mobility in frail older people are needed to maintain health and independence, yet definitive evidence of effective interventions is lacking. Our objective was to assess the impact of a multifactorial intervention on mobility-related disability in frail older people.

**Methods:**

We conducted a randomised, controlled trial with 241 frail community-dwelling older people in Sydney, Australia. Participants were classified as frail using the Cardiovascular Health Study definition, did not have severe cognitive impairment and were recently discharged from an aged care and rehabilitation service. The experimental group received a 12 month multifactorial, interdisciplinary intervention targeting identified frailty components. Two physiotherapists delivered a home exercise program targeting mobility, and coordinated management of psychological and medical conditions with other health professionals. The control group received usual care. Disability in the mobility domain was measured at baseline and at 3 and 12 months using the International Classification of Functioning, Disability and Health framework. Participation (involvement in life situations) was assessed using the Life Space Assessment and the Goal Attainment Scale. Activity (execution of mobility tasks) was measured using the 4-metre walk and self-report measures.

**Results:**

The mean age of participants was 83.3 years (SD: 5.9 years). Of the participants recruited, 216 (90%) were followed-up at 12 months. At this time point, the intervention group had significantly better scores than the control group on the Goal Attainment Scale (odds ratio 2.1; 95% confidence interval (CI) 1.3 to 3.3, *P *= 0.004) and Life Space Assessment (4.68 points, 95% CI 1.4 to 9.9, *P *= 0.005). There was no difference between groups on the global measure of participation or satisfaction with ability to get out of the house. At the activity level, the intervention group walked 0.05 m/s faster over 4 m (95% CI 0.0004 to 0.1, *P *= 0.048) than the control group, and scored higher on the Activity Measure for Post Acute Care (*P *< 0.001).

**Conclusions:**

The intervention reduced mobility-related disability in frail older people. The benefit was evident at both the participation and activity levels of mobility-related disability.

**Trial registration:**

Australia and New Zealand Clinical Trials Register (ANZCTR): ANZCTRN12608000507381.

## Background

Frailty is a common geriatric syndrome, characterised by a vulnerability to adverse health outcomes including disability, hospitalisation and institutionalisation [1,2]. As the proportion of older people rises globally, the ability to function within society at increasing age is gaining importance, and the World Health Organization has called for research into disability in the vulnerable older population [3]. The International Classification of Functioning, Disability and Health [4] uses disability as an umbrella term for problems experienced by the individual at the level of the body (impaired body structure or function), the person (activity limitation), and the person in society (participation restriction). Frail older people experience disability at each of these levels; sarcopenia and muscle weakness are prevalent [1], limitations in performing activities (for example, walking, basic activities of daily living) are common [5,6], and 80% of frail older people experience restricted participation in life roles [7]. The ability to mobilise is particularly important in this vulnerable population, as gait speed is associated with mortality [8] and dependence in older people, yet the majority of frail older people walk more slowly than average [9] and report restricted mobility in the community [7].

There is little evidence to guide interventions to prevent or reduce mobility-related disability in frail older people, particularly in terms of participation in life roles. A recent systematic review of trials evaluating the effect of exercise interventions on disability outcomes in frail older people found that only three trials used a validated definition of frailty to categorise participants [10]. Clearly defined trial populations are needed for researchers and clinicians to extrapolate study results to frail older people. Few trials have evaluated disability outcomes at a societal level in terms of participation in life roles. Mobility outcomes are predominantly evaluated at the activity level (for example, speed of gait and stair climbing, basic activities of daily living) and the few studies that have measured participation in life roles used global measures that show participation across multiple domains of life [10], so the effect of intervention on participation in the mobility domain is unknown. Owing to its multifactorial aetiology, participation may be influenced by interventions despite the presence of irreversible health conditions, impairments and activity limitations [11]. Randomised controlled trials have demonstrated that the use of mobility aids by adults with limited mobility [12] and specific training of community interactions [13] increase mobility-related participation in adults. The optimal intervention to improve mobility-related participation in life situations remains unclear however, with few controlled trials.

In a randomised controlled trial targeting the degree of frailty in frail older people [14], frailty was significantly reduced by a multifactorial interdisciplinary intervention (manuscript under review). In addition to targeting frailty, the intervention also addressed factors associated with mobility-related disability at the participation level (for example, home environment, social support, access to transport, mobility [15]) and the activity level (for example, balance, endurance). The intervention was tailored to address barriers to the mobility goal as nominated by each individual, consistent with an intervention that increased the extent of, and satisfaction with, outdoor mobility after a stroke [13].

The objective of this paper was to determine whether an interdisciplinary intervention specifically targeting frailty could reduce mobility-related disability, in terms of restricted participation in life roles and activity limitation, in community-dwelling frail older people.

## Methods

### Study design

The Frailty Intervention Trial, a prospective, parallel-group, assessor-blind, randomised, controlled, single centre trial, was undertaken from January 2008 to June 2011. The protocol for this study was registered with the Australian New Zealand Clinical Trials Registry (ANZCTRN 12608000250336) and has been published elsewhere [14]. This paper reports on the mobility-related disability outcomes of the Frailty Intervention Trial, which were registered separately (ANZCTRN 12608000507381). The primary outcomes of the trial are reported elsewhere (manuscript under review).

The protocols for the Frailty Intervention Trial and the mobility-related disability component were approved by Northern Sydney Central Coast Health Human Research Ethics Committee (1 November 2007 and 20 August 2008, respectively). Written informed consent was obtained from all participants.

### Participants

Potential participants were identified from community-dwelling patients being discharged from the hospital or community arm of the Division of Rehabilitation and Aged Care Services at Hornsby Ku-ring-gai Health Service in urban Sydney, Australia. All potential participants had completed their treatment before they were approached. Participants were eligible if they were 70 years or older; did not usually reside in a residential aged care facility; were defined as frail according to the Cardiovascular Health Study (CHS) Frailty Phenotype [1]; did not have severe cognitive impairment (defined as a Mini Mental State Examination [16] score of 18 or less); had a life expectancy exceeding 12 months (estimated by a score of three or less on a modified Implicit Illness Severity Scale) [17]; and resided in the Hornsby or Ku-ring-gai local government areas.

### Randomisation procedure

Community-dwelling older people were screened for eligibility by a research nurse (KL). Participants who gave informed consent underwent baseline assessment prior to randomisation into groups. A data analyst (NM) not involved in recruitment or assessment developed the group allocation schedule using a computer generated random number sequence and stored the list off site. Sets of permuted blocks were generated for each of two strata (three frailty criteria versus four or five frailty criteria). Block sizes of four and six were randomly arranged within blocks of 10. After baseline assessment was completed, KL was unblinded to group assignment.

### Intervention

Participants in the intervention group received a multifactorial interdisciplinary intervention targeting the CHS frailty phenotype for one year. Intervention was coordinated by two physiotherapists with extensive relevant experience and delivered by an interdisciplinary team comprising the physiotherapists, a dietician, a geriatrician, a rehabilitation physician and a nurse. Intervention was delivered primarily in the participants' homes, with additional outpatient appointments (for example, dietician, continence clinic), occupational therapy and community exercise programs offered as indicated.

The intervention is described in detail elsewhere [14]. In brief, the intervention was tailored to each participant based upon the CHS frailty criteria present in the individual at baseline (three or more of slow gait speed, weak grip strength, exhaustion, low energy expenditure and weight loss); problems identified during comprehensive geriatric evaluation; and ongoing reassessment during the 12-month intervention period. The interdisciplinary intervention was coordinated by regular case conferences and case management by the treating physiotherapist, who liaised with the participant, family, health professionals, service providers and coordinated services as indicated.

The component delivered to the intervention group that most directly targeted mobility was the 10 home-based physiotherapy sessions of 45 to 60 minutes duration. There were five sessions in the first three months after randomisation, and five sessions over the following nine months. Two of the physiotherapy sessions specifically targeted a participant-centred mobility goal. Initially developed by the participant and an assessor blinded to group, the goal was then discussed between the participant and treating physiotherapist and modified if necessary. The treating physiotherapist made a clinical assessment of the barriers to goal attainment, and then delivered interventions to target the potentially remediable barriers, such as walking capacity, social support, anxiety and community environment. The components of the goal were practised in isolation then in the target physical environment, and the degree of assistance and/or support was decreased in a safe and appropriate manner. The participation intervention protocol is available at http://www.webb.org.au.

Eight physiotherapy intervention sessions addressed the weakness, slowness and low energy expenditure CHS frailty criteria by teaching a home exercise program designed to improve mobility, increase physical activity and prevent falls (the Weight-bearing Exercise for Better Balance (WEBB) program, http://www.webb.org.au). The program was tailored to the individuals' physical impairments, prescribed three to five times per week, and reviewed and modified regularly. Appropriate equipment items such as mobility aids were also recommended.

The physiotherapist responsible for each participant recorded adherence to the study protocol and estimated a global level of adherence (in five categories) during the 12-month intervention.

Participants assigned to the control group received the usual care available to older residents of the Hornsby Ku-ring-gai area from their general practitioner and community services, which may include medical management of health conditions, allied health input, assessment of care needs and provision of care.

### Outcomes

The outcome of interest was mobility-related disability, measured at the levels of participation restriction and activity limitation. Participation was evaluated in terms of satisfaction and performance. Satisfaction with level of community access was measured using the question 'do you get out of the house as much as you would like?'[13]. This question has a dichotomous yes or no response and has demonstrated reliability and responsiveness [13]. Mobility during the preceding month was quantified in terms of distance and frequency of travel and degree of independence using the University of Alabama at Birmingham Life Space Assessment [18]. Scored on a continuous scale from 0 to 120, a higher score illustrates greater life space.

**Figure 1:**
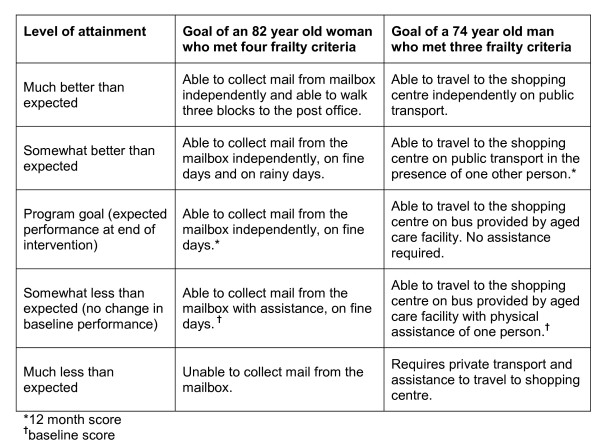
**Example of Goal Attainment Scale for two study participants**.

Achievement of individualised mobility-related participation goals was also evaluated using the Goal Attainment Scale (GAS) [19]. Recommended as a measure of relevant person-centred outcomes in frail people [20,21] and in the evaluation of complex interventions [22], the GAS has demonstrated responsiveness, adequate inter-rater reliability and concurrent validity with other rehabilitation outcome measures in older people [21,23]. Using an established methodology [24], a blinded research physiotherapist guided the participant in setting a mobility-related goal in the home environment (for example, able to weed the garden) or the community (for example, return to volunteer work) based on problems identified by the Reintegration to Normal Living Index [25]. The participant and physiotherapist established how the goal would be measured, and then the physiotherapist predicted the treatment outcomes on a five-point scale (see Figure 1). Because deterioration was plausible, baseline function was scored at the second lowest point on the scale [26]. Those who were unable to set a goal were allocated the lowest score at post-test.

Participation across multiple areas of life was measured with the Reintegration to Normal Living Index [25]. Nine of the eleven original items measure participation, covering eight dimensions of life and five International Classification of Functioning domains [27]. The nine items were included and the question phrasing was modified to increase comprehension, as validated by Daneski and colleagues [28]. The nine declarative statements were scored using a five-point scoring system (strongly disagree, disagree, neither agree nor disagree, agree, strongly agree), yielding a total score from 0 to 36, with a higher score reflective of greater participation. The tool has demonstrated reliability, content validity and construct validity [26].

Mobility performance at the activity level was measured by walking speed, measured using the 4-metre Walk Test. Self-report measures of activity were the mobility components of the Activity Measure for Post Acute Care (AMPAC) [29], which measures activity level using Item Response Theory and computer-adaptive testing, and the Nottingham Extended Activities of Daily Living Index [30].

An adverse event was defined as a medical event or injury that arose as a consequence of the trial and resulted in medical attention or restricted activities of daily living for more than two days [31].

Outcomes were assessed in participants' homes by independent, blinded nurse assessors at baseline (before randomisation) and at 3 months and 12 months after randomisation. The AMPAC was assessed via telephone at baseline and 12 months. Baseline goal setting and AMPAC assessment occurred after randomisation, by a physiotherapist blinded to group allocation.

### Blinding

Researchers who collected outcome measures and recorded data were blinded to group allocation. To ensure blinding, participants were asked not to disclose group allocation to the assessors. The participants and treating staff could not be blinded to intervention group allocation.

### Sample size

A sample size of 240 participants was chosen as it would provide 80% power to detect a 15% between-group difference in a primary outcome of the Frailty Intervention Trial - the lower extremity continuous summary performance score [32].

This sample size also provided sufficient power to detect a clinically meaningful 20% between-group difference in goal attainment and satisfaction with getting out of the house, a between-group difference of 0.1 m/s in walking speed (assuming a within-group standard deviation of 0.23) [8], and a between-group difference of 10 points in the Life Space Assessment (standard deviation of 24.7) [18]. For these calculations, we assumed an α of 0.05, non-compliance of 15% and a dropout rate of 15%.

### Statistical analysis

The primary analyses were by intention to treat [33] using Stata version 11 (College Station, TX, USA). Missing data for individual variables were imputed using multiple imputation. There was a maximum of 2% of cases missing for any variable.

To determine the effect of group allocation on continuously scored outcome measures, we examined between-group differences using linear regression models. For dichotomous outcomes, between-group differences were compared using logistic regression models. Baseline scores were entered into the linear and logistic regression models as covariates. Statistical significance was set at *P *< 0.05 and we reported the differences in percentage or mean (95% confidence interval (CI)) between the two groups at the 3-month and 12-month follow-ups.

Goal attainment was treated as an ordinal outcome; between-group difference in the distribution of goal attainment was compared using ordinal logistic regression models. The reported odds ratios (ORs) express the odds of an improved distribution of outcome in association with the intervention. To aid interpretation of the GAS, the scores were also dichotomised (goal met versus goal not met), and ORs were calculated.

We undertook a preplanned analysis to determine whether there was evidence of a differential response to the intervention for people with a cognitive impairment using a test for statistical interaction [34] (grouped by a Mini Mental State Examination score ≤ 24 or > 24)[16]. Post-hoc analyses were also conducted to test for evidence of an interaction between group and frailty severity (number of CHS criteria met, three versus more than three), and to explore the relationship between different levels of adherence (as a category variable: < 25%, 25% to 49%, 50% to 74% and ≥ 75%) and outcomes at 12-month follow-up in the intervention group.

## Results

### Participants

**Figure 2:**
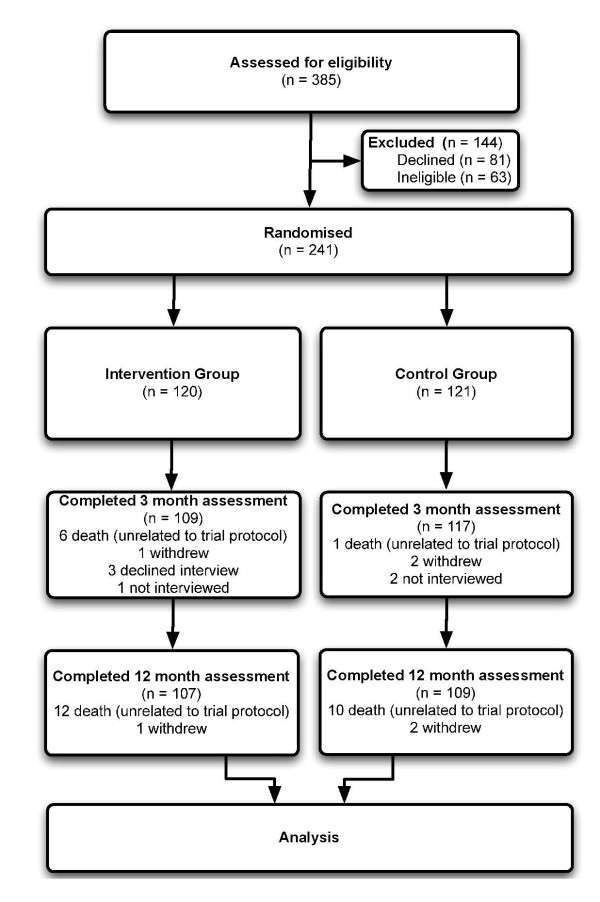
**Flow of participants through trial**.

The participant flow is summarised in Figure 2. We recruited 241 participants (68% female) with an average age of 83.3 years (SD: 5.9 years) between January 2008 and April 2010. Table 1 shows the participant characteristics at baseline. Of the 241 people randomised, 226 (94%) completed the 3-month assessment and 216 (90%) completed the 12-month assessment. The majority (22 out of 25) of losses to follow-up were due to death.

**Table 1:** Characteristics of participants in intervention and control groups at entry to study

	Intervention (n = 120)	Control (n = 121)
**Demographic factors**		
Age (years)	83.4 (5.81; 71 to 99)	83.2 (5.91; 71 to 101)
Gender, n males (%)	39 (33)	39 (32)
Lives alone, n (%)	60 (50)	51 (42)
**Health**		
Number of frailty criteria present^a^, n (%)		
3	77 (64)	79 (65)
4	33 (28)	30 (25)
5	10 (8)	12 (10)
Medical conditions^b ^(0 to 26)	7.44 (2.90; 0 to 13)	7.37 (2.58; 0 to 12)
Mini Mental Scale score^c ^(0 to 30)	26.6 (2.58; 19 to 30)	25.9 (3.14; 18 to 30)
Geriatric Depression Scale^c ^(0 to 15)	4.76 (3.18; 0 to 14)	5.06 (3.19; 0 to 14)
**Functioning**		
Walks with walking aid, n (%)	95 (79)	92 (76)
**- Activity**		
Walking speed (m/s)	0.45 (0.17; 0 to 1.00)	0.48 (0.16; 0 to 1.03)
Short Physical Performance Battery (0 to 12)	5.2 (1.89; 0 to 11)	5.74 (2.12; 0 to 12)
AMPAC, mobility scale	51.0 (8.36; 28.1 to 69.5)	54.2 (7.78 (30.8 to 69.5)
Barthel Index (0 to 100)	93.9 (11.1; 45 to 100)	92.5 (14.3; 2 to 100)
Nottingham Extended ADL Index (0 to 18)^d^	9.44 (4.1; 0 to 18)	9.13 (4.3; 1 to 18)
**- Participation**		
Gets out of the house as desired, n (%)	67 (56)	64 (53)
Life Space Assessment (0 to 120)	27.6 (12.9; 4 to 62)	30.0 (14.3; 4 to 72)
Reintegration to Normal Living Index (0 to 36)	22.1 (4.4; 5 to 31)	22.7 (3.5; 11 to 35)

Values are mean (SD, range) unless stated otherwise. ^a ^Frailty Phenotype (modified from Cardiovascular Health Study criteria) [14]; ^b ^self-reported, doctor diagnosed medical conditions; ^c ^missing data for Geriatric Depression Scale (n = 1), Mini Mental Status Examination (n = 1); ^d ^mobility component. ADL: activities of daily life; AMPAC: Activity Measure for Post Acute Care.

### Compliance with trial protocol

The median number of face-to-face intervention physiotherapy sessions in the home was 10 (range 0 to 24), and there was a median of four telephone calls to the participant. The physiotherapist delivered the WEBB program to 112 intervention participants (93%), with a median of eight sessions (range 0 to 22). The study dietician provided assessment and intervention to 60 participants (50%); 29 participants (24%) were reviewed by a geriatrician or rehabilitation physician and 4 (3%) were referred to a psychologist or psychiatrist. The physiotherapist delivered or coordinated a broad range of additional interventions, including training targeting self-selected mobility goals (61 out of 120, 51%); provision, modification or advice about equipment (48 out of 120, 40%); referral to specialist aged care services such as community transport, meal delivery, day centres, nursing, social work, occupational therapy, or teams that assess and advise on care needs (49 out of 120, 41%); and referral to medical services, such as the general practitioner, or medication-related intervention (36 out of 120, 30%).

The median global level of adherence to the intervention program, as estimated by the treating physiotherapists blinded to outcome, was 25% to 50%. Of 220 participants in the intervention group, 16 (13%) did not complete any of the intervention, 34 (29%) completed 1% to 25% of the intervention, 19 (16%) completed 26% to 50%, 25 (21%) completed 51% to 75%, and 25 (21%) completed 76% to 100%. There were 61 participants who did not complete the goal focused aspect of the intervention and the reasons for this were: inability to set a goal (17 out of 120, 14%); death before intervention commenced (3 out of 120, 3%); goal became inappropriate due to deterioration in health (35 out of 120, 30%) or the environment (for example, unsupportive family or new accommodation, 6 out of 120, 5%).

Outcome assessors remained blinded to group allocation in 97% of baseline AMPAC and GAS assessments, 100% of other baseline measures and 49% of 12-month follow-up assessments.

### Adverse events

Two intervention group participants with pre-existing musculoskeletal conditions experienced back pain severe enough to meet the study definition of an adverse event. Both resumed exercising once the prescribed exercises had been modified.

### Outcomes

#### Participation measures

The distribution of scores on the GAS at 3 months and 12 months indicated better goal attainment by the intervention group (Table 2). The OR was 4.2 (95% CI 2.6 to 7.0, *P *< 0.001) at 3 months and 2.1 (95% CI 1.3 to 3.3, *P *= 0.004) at 12 months. Dichotomised outcomes were also significantly more favourable for intervention participants than controls at 3 months (OR = 4.9 (95% CI 2.8 to 8.6, *P *< 0.001) and at 12 months; OR = 1.8 (95% CI 1.1 to 3.2, *P *= 0.03).

**Table 2:** Proportion of participants who met their self-selected participation goals at 3 months and 12 months

Outcome		Groups			
		**Intervention**		**Control**	
		
		**Month 3 (n = 111)**	**Month 12 (n = 107)**	**Month 3 (n = 117)**	**Month 12 (n = 109)**

**Goal attained**	**Degree of goal attainment**	**n (%)^a^**			

Yes	Much better than expected	9 (8)	10 (9)	0 (0)	5 (5)
	Somewhat better than expected	21 (19)	21 (20)	15 (13)	9 (8)
	Met program goal	44 (40)	23 (21)	19 (16)	25 (23)

No	Somewhat less than expected (no change in baseline performance)	27 (24)	29 (27)	47 (40)	30 (28)
	Much less than expected	10 (9)	24 (22)	36 (31)	40 (37)

^a ^May not add to 100% due to rounding

Table 3 shows the baseline, 3 month and 12 month re-test scores for the continuously measured outcomes for the intervention and control groups. The intervention group had significantly greater life space than the control group, after controlling for baseline score, at 3 months (*P *< 0.001) and at 12 months (*P *= 0.005). However, upon answering the question 'do you get out as much as you want to?', there was a trend to a poorer outcome in the intervention group, but this did not reach statistical significance (*P *= 0.17) (Table 4).

**Table 3:** Mean of intervention and control groups, and mean difference between groups for continuous outcomes

Outcomes^a^	Groups^b^						Difference between groups^c ^	
	**Intervention**			**Control**			**Intervention minus control**	
	
	**Month 0** **(n = 120)**	**Month 3** **(n = 111)**	**Month 12** **(n = 107)**	**Month 0** **(n = 121)**	**Month 3** **(n = 117)**	**Month 12** **(n = 109)**	**Month 3 adjusted for Month 0**	**Month 12 adjusted for Month 0**

Reintegration to Normal Living Index	22.1 (4.4)	25.8 (6.0)	23.1 (5.1)	22.7 (3.5)	25.1 (5.2)	22.8 (5.5)	0.92 (-0.4 to 2.2, *P *= 0.17)	0.56 (-0.7 to 1.9, *P *= 0.40)
Life Space Assessment	27.6 (12.9)	35.5 (16.1)	34.2 (16.2)	30.0 (14.3)	30.3 (13.9)	30.9 (5.5)	**6.57 (3.6 to 9.5, *P *< 0.001)**	**4.68 (1.4 to 7.9, *P *= 0.005)**
Activity Measure for Post Acute Care^d^	51.0 (8.4)	-	53.2 (7.9)	54.2 (7.8)	-	52.2 (8.80)	-	**3.16 (1.5 to 4.8, *P *< 0.001)**
NEADL	9.4 (4.1)	10.6 (4.6)	10.1 (5.2)	9.1 (4.3)	9.8 (4.7)	9.5 (4.8)	0.44 (-0.4 to 1.3, *P *= 0.3)	0.08 (-0.96 to 1.11, *P *= 0.88)
Gait speed (m/s)	0.48 (0.18)	0.50 (0.21)	0.55 (0.24)	0.50 (0.17)	0.51 (0.21)	0.50 (0.25)	0 (-0.04 to 0.04, *P *= 0.93)	**0.05 (0.0004 to 0.1, *P *= 0.048)**

^a ^Higher scores reflect better performance for all outcomes; ^b ^data presented as mean (SD); ^c ^Data presented as mean (95% confidence intervals); ^d ^not assessed at 3 months. *P*-values < 0.05 are in bold. NEADL: Nottingham Extended Activities of Daily Living Index, mobility component.

**Table 4:** Proportion of participants who were satisfied with amount of outdoor mobility at 3 months and 12 months

Outcomes	Groups^a^						Difference between groups^b^	
		
	Intervention n (%)			Control n (%)			Intervention minus control (mean (95% CI))	
		
	Month 0 (n = 120)	Month 3 (n = 1 11)	Month 12 (n = 107)	Month 0 (n = 121)	Month 3 (n = 117)	Month 12 (n = 109)	Month 3 adjusted for Month 0	Month 12 adjusted for Month 0
Do you get out as much as you want to?	67 (56)	54 (49)	47 (44)	64 (53)	57 (49)	57 (52)	-0.03 (-0.61 to 0.54; *P *= 0.91)	-0.39 (-0.95 to 0.17; *P *= 0.17)

^a ^Number (%) of participants in intervention and control groups who answered yes to the question 'do you get out as much as you want to?'; ^b ^mean (95% confidence interval) between-group difference for answer to the question 'do you get out as much as you want to?'. CI: confidence interval.

There was no clinically relevant or statistically significant between-group difference for the Reintegration to Normal Living Index at 3 months (*P *= 0.17) or 12 months (*P *= 0.40).

#### Activity measures

After 12 months, the intervention group walked 0.05 m/s faster (*P *< 0.048) over 4 metres than the control group, after controlling for baseline performance. Self-reported activity, measured using the AMPAC, was also significantly better in the intervention group compared with the control group after 12 months (4.68, 95% CI 1.4 to 9.9, *P *< 0.001). Using the Nottingham Extended Activities of Daily Living Index, the difference between groups was small and not statistically significant (*P *= 0.88).

Additional analyses were performed to explore whether participants with impaired cognition or greater frailty showed a different response to the intervention. There was no indication of an interaction between cognition and treatment group. There was a significant differential effect of the intervention on two outcomes based on baseline degree of frailty. At 3 months, the effect of the intervention on life space was greater in the less frail participants (interaction term *P *= 0.03), with a between-group difference in Life Space Assessment score of 9.0 (95% CI 5.3 to 12.7, n = 149) in the less frail group and 2.0 (95% CI -2.8 to 6.8, n = 79) in the more frail group. This differential effect was no longer significant at 12 months (interaction term *P *= 0.4). There was a greater effect of the intervention on gait speed in the more frail participants. This was not significant at 3 months (interaction term *P *= 0.9) but was significant at 12 months (interaction term *P *= 0.03) with a between-group difference of 0.13 m/s (95% CI 0.04 to 0.22, n = 76) in the more frail participants and 0.01 m/s (95% CI -0.05 to 0.07, n = 140) in the less frail.

Table 5 shows that higher adherence was significantly associated with better performance for the majority of outcomes. However, there was no significant association between adherence and three of the seven outcomes - goal attainment, AMPAC or satisfaction with getting out of the house.

**Table 5:** Outcomes at 12 months by adherence to intervention, adjusted means (95%CI) or odds ratio (95%CI)

Outcome	Statistic	Adherence				*P*
		**< 25%** **(n = 42)**	**25% to 49%** **(n = 17)**	**50% to 74%** **(n = 23)**	**75% to100%** **(n = 24)**	
Reintegration to Normal Living Index	Mean^a^	22.0 (20.7 to 23.3)	22.9 (20.8 to 24.9)	23.8 (22.0 to 25.5)	24.8 (23.1 to 26.5)	< 0.01^c^
Life Space Assessment	Mean^a^	30.5 (26.7 to 34.2)	33.9 (27.8 to 40.1)	36.7 (31.6 to 41.8)	38.7 (33.7 to 43.8)	< 0.01^c^
Activity Measure for Post Acute Care	Mean^a^	52.1 (50.2 to 54.0)	51.1 (48.0 to 54.2)	55.9 (53.4 to 58.5)	54.4 (51.8 to 56.9)	0.05^d^
Nottingham Extended Activities of Daily Living index	Mean^a^	8.48 (7.23 to 9.74)	9.61 (7.58 to 11.6)	10.7 (8.99 to 12.4)	12.7 (11.1 to 14.4)	< 0.001^c^
Gait speed	Mean^a^	0.48 (0.42 to 0.54)	0.56 (0.47 to 0.65)	0.58 (0.51 to 0.66)	0.62 (0.55 to 0.69)	< 0.01^c^
Satisfaction with outdoor mobility	Odds ratio^a^	1.00	2.05 (0.57 to 7.32)	2.62 (0.84 to 8.12)	2.35 (0.75 to 7.36)	0.29^e^
Goal Attainment Scale (degree of goal attainment)	Odds ratio^b^	1.00	1.29 (0.45 to 3.68)	3.24 (1.28 to 8.23)	2.06 (0.82 to 5.19)	0.08^e^

^a ^Adjusted for age, gender, living alone, number of frailty criteria, Mini Mental State Examination score and baseline values; ^b ^odds ratio from ordinal logistic regression model adjusting for age, gender, living alone, number of frailty criteria and Mini Mental State Examination score; ^c ^*F *Test: test for trend in means across ordered groups (degrees of freedom = 1), treating groups as ordered (continuous) variable; ^d ^*F *Test: test means between four groups (degrees of freedom = 3), treating groups as a factor; ^e ^likelihood test.

## Discussion

A 12-month multifactorial intervention targeting frailty was more effective than usual care in reducing mobility-related disability in community-dwelling frail older people. At the participation level, gains were present at both 3 and 12 months after intervention commenced. The intervention also increased mobility outcomes at the activity level at 12 months and was associated with minor adverse events. However, significant improvements were not detected using several outcome measures, and some statistically significant improvements may not have been clinically meaningful. To our knowledge, this is the first randomised trial to evaluate the effect of an intervention targeting frailty on mobility-related disability in older people who met specified frailty criteria.

Our sample of frail older people had poor mobility at baseline. They had an average of seven medical conditions and walked at one quarter the speed of healthy older people [35]. Most had recently been discharged from an aged care and rehabilitation service and almost half did not get out of the house as much as they wanted to. At 12 months, we found the distribution of goal attainment on the GAS was significantly more favourable in the intervention group (OR 2.1, 95% CI 1.3 to 3, *P *= 0.004). We acknowledge that the GAS needs to be interpreted with caution however, as the non-linearity of ordinal scores at the margins of the score range (37% of scores at 12 months) can generate exaggerated change scores [36]. The intervention as delivered also significantly increased life space; how often people mobilised in the home and community, how far they went, and their degree of independence. The improvement reached statistical, but not clinical, significance [18]. Despite an increase in the extent of mobility, the intervention group showed a non-significant trend toward less satisfaction with their ability to get out of the house.

Interestingly, although participation in the mobility domain increased, there was no effect on the global measure of participation. This may be because the intervention focused primarily on the components of frailty, principally mobility, and targeted mobility-related participation goals. There are few trials measuring participation in frail older people with which to compare our findings. Previous systematic reviews of the effect of exercise and trials of geriatric evaluation and management on functional outcomes in frail older adults [10,37,38] have reported function primarily in terms of body structure and/or function and activity, whilst participation outcomes have been largely unreported.

There were statistically significant between-group differences for two of the three measures of the activity aspect of disability. At 12 months, the intervention group performed better on the AMPAC and walking speed. The effect of the intervention on activity outcomes is consistent with previous studies in frail older people, which indicate gait speed and composite activity measures may improve with regular multicomponent training over a prolonged period [10]. The mean increase in walking speed of 0.05 m/s was at the suggested cut-off for a small meaningful change in a sample of older people with a higher level of functioning [39], so may be clinically significant in this comparatively frail group. Also, given the association between gait speed and survival [8], the significant improvement is noteworthy in this vulnerable population. In the absence of consensus on the clinically meaningful difference in AMPAC score in this population, we applied Norman and colleagues' criteria that the minimally important difference can be estimated as half the baseline standard deviation of raw scores [40]. Although the mean between-group difference in AMPAC score was statistically significant, it was less than the minimally important difference. The relatively high degree of difficulty of the mobility tasks in the Nottingham Extended Activities of Daily Living Index mobility outcome may account for the lack of between-group difference in this outcome.

There appears to be a significant differential effect of the intervention on the walking speed and life space aspects of disability according to degree of frailty at baseline. The intervention had a lesser effect on walking speed and a greater effect on life space in participants who met three frailty criteria compared with the participants who met four or five criteria. This effect should, however, be interpreted with caution and requires further investigation [34]. Participants with higher levels of adherence to the intervention had better outcomes after adjusting for possible confounders. This may indicate a dose-response effect of the intervention, but we acknowledge the potential biases associated with analysis of such adherence data [41].

It is not possible to determine which aspect of the multifactorial intervention increased participation in the mobility domain. There was a significant between-group difference in goal attainment, despite only half of intervention participants receiving the two intervention sessions specifically targeting participation goals. Participation is associated with multiple factors, including degree of frailty, mood, strength and walking speed [7,42], and it is feasible that improvement in such elements contributed to gains in participation in life roles. Further research is required to understand which components of an intervention are required to improve participation and how participation can be improved across multiple areas of life.

The strengths of our study were the use of a validated definition of frailty, broad generalisability to recently hospitalised community-dwelling frail older people, the small losses to follow-up that were similar in both groups, and adherence to sound trial design and methodology. Also, the intervention - delivered in the setting of an existing health service by an interdisciplinary team experienced in aged care - resembles that deliverable in clinical practice. We acknowledge, however, that the study had limitations. First, participants could not be blinded to group allocation, which is a potential source of bias due to possible differential reporting of self-report outcomes such as goal attainment. Second, adherence with the program was variable; however, this is likely to be the case with treatment programs delivered to frail populations in the clinical setting, where health, physical and social needs fluctuate. Third, as there was no frequency-matched social intervention for the control group, we cannot exclude the impact of social aspects of the program on the difference between groups. Finally, although we attempted blinding of outcome assessors, 123 participants (51%) inadvertently disclosed their group status (that is, mentioned their exercise program) to research personnel at the 12-month follow-up.

Understanding disability in frail older people is hindered by the infrequent use of validated diagnoses, the systematic exclusion of frail older people from trials [43] and the often narrow conceptualisation of disability. This paper adds to the evidence that community-dwelling frail older people have the potential for functional improvement in both the participation and activity domains, through multifactorial intervention. Interventions that reduce disability in the frail population have the potential to impact on morbidity, hospitalisation and admission to residential care facilities, along with the associated costs to government and society.

## Conclusions

For frail older people residing in the community, a 12-month multifactorial interdisciplinary program targeting frailty was more effective than usual care in reducing mobility-related disability at the participation and activity levels. The intervention increased walking speed, the extent of mobility in the home and community and the likelihood of meeting participation goals, however some significant improvements may not be clinically meaningful and neither satisfaction with ability to leave the home nor participation in broader aspects of life were significantly improved. It is recommended that future studies in frail older people measure mobility at both the participation and activity levels. Future research should also include a longer follow-up period to determine if the benefits of the intervention are maintained after 12 months.

## Abbreviations

AMPAC: Activity Measure for Post Acute Care; CHS: Cardiovascular Health Study; CI: confidence interval; GAS: Goal Attainment Scale; OR: odds ratio.

## Competing interests

The authors declare that they have no competing interests.

## Authors' contributions

NF contributed to the design and coordination of the mobility-related disability project, delivery of the intervention, collection, analysis and interpretation of the data and drafted the manuscript. CS, SEK, SRL and IDC were the chief investigators on the Frailty Intervention Trial. CS contributed to study design, analysis and interpretation of the mobility-related disability data. SRL contributed to the study design and interpretation of the data. KL contributed to study design, recruitment, data management and interpretation of the data. SEK and IDC conceived the Frailty Intervention Trial, contributed to the design of the mobility-related disability component, delivery of the intervention and interpretation of data. All authors contributed to revisions and approved the final manuscript.

## Authors' information

NF is a Physiotherapist and a PhD candidate investigating participation in life roles by frail older people.

CS is a Senior Research Fellow at the George Institute for Global Health, The University of Sydney. She is an Honorary Senior Research Associate at Neuroscience Research Australia, The University of New South Wales.

SEK is a Geriatrician and Clinical Director of Rehabilitation and Aged Care Services at Hornsby Ku-ring-gai Hospital.

SRL is a Senior Principal Research Fellow at Neuroscience Research Australia, The University of New South Wales, Sydney.

KL is a Registered Nurse and Research Consultant, who coordinated recruitment and data management on the Frailty Intervention Trial.

IDC is a Consultant Physician in Rehabilitation Medicine. He is the first named investigator for the National Health and Medical Research Council Program Grant 'Transition Care: Innovation and Evidence' of which the Frailty Intervention Trial is one of a number of studies.

## Pre-publication history

The pre-publication history for this paper can be accessed here:

http://www.biomedcentral.com/1741-7015/10/120/prepub
